# A Typology of Patients Based on Decision-Making Styles: Cross-Sectional Survey Study

**DOI:** 10.2196/15332

**Published:** 2019-11-20

**Authors:** Mary Anne FitzPatrick, Alexandra Claudia Hess, Lynn Sudbury-Riley, Peter Johannes Schulz

**Affiliations:** 1 School of Management and Marketing Waikato Management School University of Waikato Hamilton New Zealand; 2 School of Communication, Journalism and Marketing Massey University Auckland New Zealand; 3 Management School University of Liverpool Liverpool United Kingdom; 4 Institute of Communication and Health Faculty of Communication Science University of Lugano Lugano Switzerland

**Keywords:** internet, online health information, patient decision making, patient-practitioner interaction, patient segments, patient typology, baby boomers, patient education

## Abstract

**Background:**

Although previous research shows broad differences in the impact of online health information on patient-practitioner decision making, specific research is required to identify and conceptualize patient decision-making styles related to the use of online health information and to differentiate segments according to the influence of online information on patient decision making and interactions with health professionals.

**Objective:**

This study aimed to investigate patients’ decision making in relation to online health information and interactions with health care practitioners. We also aimed to present a typology of patients based on significant differences in their decision making.

**Methods:**

We applied a large-scale cross-sectional research design using a survey. Data, generated using a questionnaire that was administered by companies specializing in providing online panels, were collected from random samples of baby boomers in the United Kingdom, the United States, and New Zealand. The total sample comprised 996 baby boomers born between 1946 and 1964, who had used the internet in the previous 6 months to search for and share health-related information. Data were analyzed using hierarchical cluster analysis and confirmatory factor analysis, as well as one-way analysis of variance, chi-square tests, and paired sample *t* tests.

**Results:**

Analyses identified 3 key decision-making styles that served as the base for 4 unique and stable segments of patients with distinctive decision-making styles: the Collaborators (229/996, 23.0%), the Autonomous-Collaborators (385/996, 38.7%), the Assertive-Collaborators (111/996, 11.1%), and the Passives (271/996, 27.2%). Profiles were further developed for these segments according to key differences in the online health information behavior, demographics, and interactional behaviors of patients. The typology demonstrates that collaborative decision making is dominant among patients either in its pure form or in combination with autonomous or assertive decision making. In other words, most patients (725/996, 72.8%) show significant collaboration in their decision making with health care professionals. However, at times, patients in the combination Autonomous-Collaborative segment prefer to exercise individual autonomy in their decision making, and those in the combination Assertive-Collaborative segment prefer to be assertive with health professionals. Finally, this study shows that a substantial number of patients adopt a distinctly passive decision-making style (271/996, 27.2%).

**Conclusions:**

The patient typology provides a framework for distinguishing practice-relevant and addressable segments with important implications for health care practitioners, including better-targeted communication programs for patients and more successful outcomes for health care services in the long term.

## Introduction

### Background

Within the patient-practitioner interaction, decision making is a critical interactional process that can be affected markedly by patient-sourced online health information. Recognized as an ethical imperative in terms of respect for patient preferences, values, and circumstances [1] and as a means to better manage scarce health care resources [2], decision making has crucial consequences ranging from patient health outcomes [3] to health care costs [4]. Previous research has found broad differences in the impact of online health information on decision making [5-7]. However, researchers have paid little attention to identifying and conceptualizing specific patient decision-making styles associated with online health information. In addition, no research has yet segmented or profiled patient groups based on their decision-making styles.

### Patient Decision Making and Online Health Information

Decision making is a specific interactional process influenced by patient-sourced online health information [5,6,8-10]. Of particular relevance to this research are previous studies that detail a variety of decision-making behaviors by internet-informed patients; in particular, behaviors involving health care professionals. Previous research confirms that online health information can empower patients to be more active participants in decision-making with practitioners [7,11]. Such studies show that internet-informed patients can possess both knowledge and treatment preferences before they interact with practitioners, making them better equipped to take a fuller and more participatory role in decision making.

Shared decision making, the collaborative process in which available information is actively shared and health care decisions are made jointly by patients and practitioners, is “the focal point of a whole set of interlinking shifts and reforms related to the changing roles and relationships between doctors and patients” [12]. Grounded generally in broader principles of consumer rights, shared decision making in the context of patient-practitioner interactions reflects both a global upsurge in need for chronic care [13] and the shift from professional paternalism in health care services [14]. However, several scholars caution that shared decision making is an ideal [15], a viewpoint supported by evidence of a variety of patient decision-making experiences that do not conform to the ideal of participative collaboration. For example, patients can be reluctant to collaborate with practitioners either for fear of being labeled *difficult* and consequently less likely to receive quality care or because they perceive that active participation would threaten the relationship with their practitioner [15]. Previous research also finds patients can be uncomfortable asking questions of their practitioners because they prefer to conform to the passive role for patient or to defer to the practitioner as the authority [15,16].

In addition, patients can find it difficult to participate in collaborative decision making when their health literacy is low [17], when they lack the necessary evaluative skills to work through the decision-making process [18], or when they are facing a serious health condition [14]. Shared decision making is likely to be especially problematic for chronic patients who represent a sizeable—and growing—proportion of the population and are expected to take more responsibility themselves for managing their illnesses [17]. Patients with chronic conditions have more frequent consultations [19] and source complex health information from multiple sources, including changing networks of different health professionals [20].

Online health information also enables patients to self-diagnose and/or self-treat without visiting a practitioner. Prior research indicates that making internet-informed decisions independently of a practitioner can improve patients’ self-efficacy and reduce unnecessary visits to practitioners—changes that are in line with trends toward increased patient responsibility and self-management of health [17,19]. In contrast, other scholars draw attention to the increased health risks for patients who decide to manage their health care independently [6,21].

Furthermore, patients can introduce online health information that is opposite to or actively challenges practitioners’ diagnoses, prognoses, treatment, and advice. This set of patients’ behaviors encompasses online health information that patients misunderstand but insist on, as well as information that patients interpret correctly but that conflicts with practitioners’ opinions or threatens their professional authority [22]. These behaviors can result in inappropriate use of health care resources when the practitioner accedes to requests for unsuitable tests or treatments. Practitioner resistance, on the contrary, can result in patient dissatisfaction linked to nonadherence [23], seeking a second opinion (offline or online), switching health care providers, or changing the treatment plan [7].

Other studies confirm some patients decide not to share the online health information they have acquired with practitioners [6,24,25]. Having informed themselves before the interaction, nonsharing patients can feel more empowered in their decision making because they are better able both to understand what the practitioner says [22] and to trust the practitioner when the online information verifies the practitioner’s explanation [26].

### Styles of Decision Making

In summary, previous research has found important differences in patient behavior involving online health information and decision making with practitioners. On the basis of the literature, this study proposed the following 3 distinguishable decision-making styles to be examined further in exploratory research:

- Collaborative decision making: following this style, the patient uses online health information to collaborate with the health professional and work as an active partner in making decisions about treatment and management.
- Autonomous decision making: this style applies to the patient who uses online health information to make autonomous decisions without involving the health care practitioner.
- Assertive decision making: the patient using this style draws on online information to assert his or her own preferences for treatment and management and/or to oppose practitioner advice in decision making.

### Rationale

This research focused on investigating the decision-making behavior of baby boomer patients born between 1946 and 1964, an age cohort of substantial size. Baby boomers will place greater demands on health care professionals and exert considerable pressure on health care systems as longevity increases, rates of age-related and chronic conditions worsen, and lifetime health care costs escalate [27]. An increasing number of baby boomers uses the internet to search for and share health information [28] and assume a more active role in their own health care compared with preceding generations [29]. These changing patterns in baby boomers’ health care consumption highlight an urgent need to better understand their patient behavior—in particular, their decision making—as heavy users of health care services. Such knowledge will provide a sound base for implementing health care programs that better meet the needs of different groups of internet-informed patients.

### Objectives

The overall aim of this research was to address gaps in knowledge on patient decision making. We set out with the following 2 specific objectives: (1) to investigate the decision-making styles of patients in relation to online health information and interactions with health care practitioners and (2) to develop a typology of patients based on similarities and differences in those decision-making styles.

From these objectives, we then formulated the following research questions: (1) What are patient decision-making styles in relation to online health information and their health care practitioners? and (2) How can internet-informed patients be segmented and described based on their decision-making styles?

## Methods

### Research Design

To answer the research questions and achieve the study objectives, we applied a large-scale cross-sectional research design using a survey to generate quantitative data. The survey was conducted in the United Kingdom, the United States, and New Zealand to enable cross-national segmentation of patients according to their decision making, online health information, and interactional behaviors. We followed the research practices of other researchers conducting cross-national segmentation, who contend that because of globalization, segmentation should take account of commonalities across countries as well as differences within countries [30-34]. The rapid pace of globalization and the spread of a global culture fueled by the internet [30,34] endorse cross-national segmentation as an appropriate approach to identifying between-country segments of internet-informed patients with similar decision-making styles. We measured the patient behaviors at the individual level (following Steenkamp and Ter Hofstede [35]) rather than at the country level and worked with a single dataset, as is standard in cross-national segmentation, to develop a segmentation scheme that would partition respondents from the 3 countries into groups of patients with similar decision-making styles. We used a *country of residence* variable to account for differences by country, enabling fuller descriptions and better characterization of patient segments. Ethics committees of the University of Waikato, the University of Liverpool, and the University of Lugano approved the research before it commenced.

### Questionnaire Development

The first step involved developing items for the proposed patient decision-making styles associated with online health information seeking (collaborative, autonomous, and assertive) and patient-practitioner interactions. We adopted well-established procedures [36,37] to develop a multidimensional measure of patient decision-making styles. For example, following Boateng et al [37], we based item generation on a review of the literature and existing scales, combined with data from focus groups (3 groups of 6 participants each: 1 mixed gender group, 1 female, and 1 male) and semistructured in-depth interviews (8 participants).

A total of 22 items was generated relating to patient-sourced online health information and decision making involving the health care practitioner. To ensure content validity, other faculty members experienced in the subject area (ie, expert judges) rated how well each of the items related to the 3 proposed decision-making styles. The resulting instrument was then subjected to 3 rounds of pretesting for item reduction and scale purification purposes. Together, these processes resulted in the retention of 10 items: 3 collaborative items, 3 autonomous items, and 4 assertive items. All 10 items appear in Table 1 (8 items appear within the table, and 2 items are listed in the table footnote). The final 10 items relating to patient decision-making styles used a Likert agreement scale ranging from 1 to 5, in line with researchers who employ this scale as an ordinal approximation of a continuous variable without compromising data analysis [38-41].

**Table 1:** Items and factor loadings of exploratory factor analysis.

Patient behavior item^a^	Patient decision-making style		
	Collaborative	Autonomous	Assertive
I have used online health information to ask questions of my health professional(s)	0.75	—^b^	—
I have had my diagnosis confirmed by my health professional	0.78	—	—
I have sought help from a health professional	0.86	—	—
I have changed the treatment recommended by a health professional	—	—	0.82
I have refused or discontinued treatment recommended by my health professional	—	—	0.83
I have changed from one health professional to another	—	—	0.75
I have tried to treat a health condition or disease without help from a health professional	—	0.83	—
I have tried to diagnose a health condition or disease I or someone else might have	—	0.84	—
Eigenvalue	3.591	1.685	3.395

^a^On the basis of split loadings and reliability analysis, the following 2 items were removed: “I have sought a second opinion from another health professional” (assertive) and “I didn’t need to visit a health professional” (autonomous).

^b^Loadings of less than 0.5 are not shown to improve readability.

Following common practice in cluster analysis, our questionnaire design went beyond investigation of the 3 decision-making styles (which we refer to as *active* variables) to include other variables (which we refer to as *passive* variables); thus, we could identify practice-relevant and addressable segments. For example, we included frequency of visits to a health professional (measured using items based on questions in the Health Information National Trends Survey 2014; refer [42]) as well as variables relating to patients’ online behavior and online information outcomes (see Multimedia Appendix 1 for more details). In addition, we used a range of other demographic variables, including *country of residence* to allow for differences by country in adopting different decision-making styles (see Multimedia Appendix 2 for more details). We used health-related variables such as living with chronic health problems and electronic health (eHealth) literacy (measured using items based on questions in Pew Research Center’s *The Internet and Health* questionnaire [43]). The questionnaire provided a *not applicable* response for suitable questions to ensure there would be no missing data.

To check validity and reliability of the final items, we again asked the 6 experts to evaluate how well the constructs we wanted to measure were represented by relevant items in the questionnaire. Then, we further confirmed the validity and reliability of the survey via a pilot test of the questionnaire for readability and understandability (n=64). The questionnaire was prepared and administered in English to avoid problems translating questions and issues with conceptual equivalence [30].

### Procedure

We outsourced the recruitment of baby boomer patients to external commercial organizations that specialize in online panels [44]. The organizations were instructed to sample respondents born between 1946 and 1964 who had used the internet in the previous 6 months to search for and share health-related information. The organizations were directed to distribute the link to the online questionnaire to a representative sample in terms of gender, ethnicity, education, income, and location. For sampling, the companies relied on registered members in their databases who receive compensation (points based) for participating in individual surveys. Data were collected over a 4-month period in the United Kingdom, the United States, and New Zealand to facilitate cross-national segmentation. A total sample of 996 (the United Kingdom, n=407; the United States, n=313; and New Zealand, n=276) respondents completed usable questionnaires with no missing data.

### Data Analysis

To enable us to explore the existence of a common segmentation scheme across the United Kingdom, the United States, and New Zealand, data from the 3 countries were aggregated for analysis. First, we conducted a set of preliminary analyses, including descriptive data analysis, identification of outliers, and nonnormality checks. Then, to empirically test our 3 decision-making styles, we conducted an exploratory factor analysis, followed by a confirmatory factor analysis (CFA). Following this, based on the 3 decision-making styles (active variables), we conducted a cluster analysis. We began by employing a hierarchical cluster procedure using Ward method and measured the distance between cases using the square of the Euclidean distance. This procedure was followed by a cluster analysis using k-means. To develop fuller profiles of each segment (following Barnes et al [45]), we used descriptive characteristics including online information outcome, health-related domain, online domain, and demographic variables as passive variables (see Multimedia Appendices 2-5 for details). We used one-way analysis of variance, chi-square tests, and paired sample *t* tests to describe our segments. Data analysis resulted in a typology comprising 4 distinct patient segments with multidimensional profiles, which we present in the following sections.

## Results

### Sample Description

The sample contained slightly more females than males (499/996, 50.1%). In terms of ethnicity, the majority of respondents identified themselves as white or Caucasian (872/996, 87.6% of total sample; 389/407, 95.6% of UK respondents; 249/313, 79.6% of US respondents; and 234/276, 84.8% of New Zealand respondents). In terms of educational levels, most respondents had completed college and practical, technical, or occupational training (350/996, 35.1%), followed by 33% (329/996) with a university degree, and 30.6% (305/996) reporting high school as the highest educational level (see Table 2 for details). The total sample appears to be relatively homogenous on these demographic details, and the results should be interpreted in light of this.

**Table 2:** Sample characteristics by country (N=996).

Characteristics		United Kingdom (n=407)	United States (n=313)	New Zealand (n=276)	Total (N=996)
**Sex, n (%)**					
	Male	193 (47.4)	163 (52.1)	141 (51.1)	497 (49.9)
	Female	214 (52.6)	150 (47.9)	135 (48.9)	499 (50.1)
**Age (years), n (%)**					
	46-49	40 (9.8)	55 (17.6)	72 (26.1)	167 (16.8)
	50-54	124 (30.5)	72 (23.0)	64 (23.2)	260 (26.1)
	55-59	95 (23.3)	92 (29.4)	69 (25.0)	256 (25.7)
	60-64	148 (36.4)	94 (30)	25.7 (52.1)	313 (31.4)
**Ethnicity, n (%)**					
	White	389 (95.6)	249 (79.6)	234 (84.8)	872 (87.55)
	Non-white	18 (94.4)	64 (20.4)	42 (15.2)	124 (12.45)
**Educational attainment, n (%)**					
	Less than high school	3 (0.7)	8 (2.6)	0 (0.0)	12 (1.2)
	High school	158 (38.8)	59 (18.8)	89 (32.2)	305 (30.6)
	College/practical/technical/occupational	148 (36.4)	101 (32.3)	101 (36.6)	350 (35.1)
	University degree	98 (24.1)	145 (46.3)	86 (31.2)	329 (33.0)

### Patient Decision-Making Styles

EFA supported the 3 decision-making styles indicated by the literature and exploratory research (see Table 1 for factor loadings). On the basis of split loadings and reliability analysis, 2 of the 10 items were eliminated. The total variance extracted by the 3 factors was 72%, the Bartlett test of sphericity was significant with *P*<.001, and the Kaiser-Meyer-Olkin measure was 0.78, indicating construct validity. The Cronbach *alpha* values of the remaining 8 items were of an acceptable standard for the study’s item structure (collaborative *alpha*=.77, autonomous *alpha*=.69, and assertive *alpha*=.77; refer to the study by Loewenthal [46]).

Furthermore, a CFA of the 8 items (applying AMOS 24 testing for convergent and discriminant validity) indicated that the 3 measurement models provided a good model fit (see Table 3). Factor loadings of all items in the 3 models were higher than 0.6. Average variance extracted (AVE) was greater than 0.5, and compositional reliability (CR) was above 0.6. Thus, together, AVE and CR satisfied the requirement for convergent validity [32]. Finally, AVEs were higher than the squared correlations between constructs, thus supporting the discriminant validity of the model (see Table 3 for details) [47].

**Table 3:** Descriptive statistics and convergent and discriminant validity results.

Construct^a^	Mean based on total sample (SD)	Parameter estimates of confirmatory factor analysis (range)	Compositional reliability	Average variance extracted	Correlation/discriminant validation			Statistics						
					Collaborative	Autonomous	Assertive	χ^2^ (df=15)	*P* value	Goodness-of-fit index^b^	Comparative fit index^c^	Normed fit index^d^	Tucker-Lewis index^e^	Root mean square error of approximation^f^

Collaborative	2.83 (0.94)	0.61-1.0	0.836	0.64	—^g^	0.1	0.16	120.8	<.001	0.97	0.96	0.95	0.92	0.08
Autonomous	3.34 (1.0)	0.68-0.77	0.69	0.528	0.31	—	0.43	—	—	—	—	—	—	—
Assertive	3.81 (0.84)	0.61-0.82	0.783	0.549	0.4	0.18	—	—	—	—	—	—	—	—

^a^The calculated values of the square correlation coefficient between all possible pairs of constructs are presented in the upper triangle of the matrix. Correlations between all pairs of constructs are presented in the lower triangles of the matrix (*P*<.01).

^b^Values higher than 0.95 indicate better model fit [48].

^c^Values greater than 0.95 indicate a good fit [49].

^d^Values greater than 0.95 indicate a good fit.

^e^Values greater than 0.9 are considered a satisfactory fit [50].

^f^Values between 0.05 to 0.1 are considered a fair fit [51], with values below 0.08 acceptable [50].

^g^Not applicable.

### Patient Segments

Initial analysis of data generated by the survey focused on the validation and interpretation of segments distinguished by statistical differences in the decision making of segment members. To determine the number of segments, first, a hierarchical cluster procedure (Ward method) was used, and the square of the Euclidean distance measured the distance between cases. Moreover, 3-, 4-, 5-, and 6-cluster solutions were explored. To determine the number of groups, the researchers considered the dendrograms and the distance at which each cluster was formed, profiled each cluster, and applied practical judgment and theoretical foundations [52]. All these indicators suggested that the 4-cluster solution was the most acceptable. Next, a k-means clustering analysis of the 4-cluster solution was performed. A pairwise cluster validation revealed that all 3 patient decision-making styles were significant in distinguishing between the 4 clusters. A pairwise analysis of the differences between the clusters revealed that, on average, 83% of the individual pairwise differences were significant at *P*<.05, showing the 4 clusters obtained using the patient decision-making styles are unique and stable. On the basis of analysis, we refer to the clusters as *segments* of patients labeled the Collaborators, the Autonomous-Collaborators, the Assertive-Collaborators, and the Passives.

Thus, as expected, we did find the 3 decision-making styles we derived from previous research. Furthermore, based on our cluster analysis, we found that the majority of patients adopt a Collaborative decision-making style (as we had proposed), including 2 segments that adopt combination Autonomous-Collaborator and Assertive-Collaborator decision-making. We also found 1 group of patients that does not adopt collaborative decision making: the segment of Passive patients (see Table 4 for details of each segment).

Our data analysis to this point showed that patients could be segmented into 4 distinct segments using the 3 decision-making style variables as active variables (see Table 4). In the next step, we used passive variables in our analysis, which allowed us to take account of differences (eg, country of residence) to provide fuller, more nuanced profiles of the 4 decision-making segments. Multimedia Appendices 2 and 3 provide descriptions of the segments by relevant variables.

The following subsections detail each segment. Full profiles of each are presented in the Discussion section.

**Table 4:** Final cluster solution and analysis of results.

Decision-making style	Total sample	Segment 1^a^	Segment 2	Segment 3	Segment 4	*F* test	*P* value
		Collaborators	Autonomous-Collaborators	Assertive-Collaborators	Passives		
Collaborative, mean (SD)	3.17 (0.94)	3.7 (0.55)	3.5 (0.65)	3.82 (0.59)	1.99 (0.55)	494.107 (995)	<.001
Autonomous, mean (SD)	2.61 (1.00)	1.98 (0.65)	3.56 (0.56)	2.28 (0.62)	1.96 (0.79)	435.242 (995)	<.001
Assertive, mean (SD)	2.2 (0.84)	1.66 (0.46)	2.64 (0.75)	3.13 (0.57)	1.63 (0.52)	287.477 (995)	<.001
Cluster size	—^b^	229	385	111	271	—	—
Percentage of respondents	—	23.0	38.7	11.1	27.2	—	—

^a^All segment means are significant at the .001 level, and 83% of the pairwise comparison is significant at *P*<.05 level. All variables are coded on a 5-point scale, with 1 for strongly disagree to 5 for strongly agree.

^b^Not applicable.

#### Segment 1: Collaborators

This segment describes 23.0% (229/996) of respondents, making it the third largest of the 4 segments. This research finds patients in segment 1 report that they rely significantly more on their health professional than the internet for health-related information. However, Collaborative decision makers state that as a result of searching online for health information, they can communicate with their health professionals significantly better compared with Autonomous-Collaborators (*P*<.05; see Multimedia Appendix 3 for more details). Collaborators, who are moderately eHealth literate (slightly lower than patients in the autonomous-collaborator segment with *P*=.08, that is, marginally significant; see Multimedia Appendix 4 for details), talk significantly more to practitioners about problems with the online health information they have accessed than Autonomous-Collaborator patients (*P*<.05; see Multimedia Appendix 5 for more details). In addition, Collaborators typically use the internet less to interact with others online about health-related matters than the combination Autonomous-Collaborators and Assertive-Collaborators (all *P* values <.05; see Multimedia Appendix 5 for more details). For example, collaborators go online less frequently to read others’ health experiences, to share their own personal health experiences, or to post a comment or review online about a health-related product, service, or person.

Likely to be living with a chronic health problem (*P*<.05; see Multimedia Appendix 4 for more details), people in this segment visit their health care practitioners more often compared with Autonomous-Collaborators (*P*<.05; see Multimedia Appendix 3 for more details). This segment is not dominated by any particular nationality, with US citizens representing 34.9% (80/229), UK citizens representing 31.9% (73/229), and NZ citizens representing 33.2% (76/229). Segment 1 patients are most likely to be retired and receiving government welfare (eg, pension). This segment, which has the largest number of widowed respondents (19/53), includes a cross-section of ages across the baby boomer cohort (all *P* values <.05; see Multimedia Appendix 2 for more details).

#### Segment 2: Autonomous-Collaborators

The largest single group at 38.7% (385/996), the Autonomous-Collaborator segment, is made up of patients who exhibit a combination of both collaborative and autonomous styles in their decision making with health care practitioners. Autonomous-Collaborator patients differ significantly from Collaborators in that interaction and support from others online are more important, read others’ commentaries about health online, and post more questions and reviews online (all *P* values <.05; see Multimedia Appendix 5 for more details). Patients in this segment frequently go online in an attempt to diagnose a health condition themselves. In fact, autonomous-collaborators report that they rely significantly more on the internet than the health professional for health-related information (*P*<.05). When they encounter a problem with internet-sourced health information, Autonomous-Collaborators talk about it with friends and/or someone online significantly more than Collaborators (*P*<.05; see Multimedia Appendix 5 for more details). This research shows that patients who use a combination of autonomous and collaborative decision-making styles in interactions with health care practitioners are slightly more eHealth literate than the pure Collaborator (*P*=.08; ie, marginally significant; see Multimedia Appendix 4 for more details). They are neither more nor less likely to be living with a chronic health problem (see Multimedia Appendix 4 for more details), and they make a moderate number of visits to health care practitioners (significantly less compared with Collaborators and Assertive-Collaborators; *P*<.05; see Multimedia Appendix 3 for more details).

In terms of other outcome variables, this segment reports slightly improved communication and relationship quality with their practitioners, yet less overall compared with Collaborator and Assertive-Collaborator patients (*P*<.05 difference for communication; *P*=0.05 difference in relationship quality between Autonomous-Collaborator and Assertive-Collaborator; see Multimedia Appendix 3 for more details). This Autonomous-Collaborator segment is comprised mainly of UK citizens; with 49.1% (189/385) of respondents, this is the largest percentage of any nationality in the research, whereas New Zealand (81/385, 21%) and US (115/385, 29.9%) respondents represent significantly smaller national groups in the segment. Segment 2 patients are most likely to have never married. This segment is made up predominantly of patients born between 1960 and 1964, the Fourth Wave and youngest of the baby boomer generation (*P*<.05; see Multimedia Appendix 5 for more details).

#### Segment 3: Assertive-Collaborators

This segment, the smallest at 11.1% (111/996) of the sample, comprises patients characterized by a distinctive combination of collaborative and assertive decision-making styles. Assertive-Collaborators have the highest frequency of online searches for health-related information overall.

Data analysis reveals that Assertive-Collaborator patients are differentiated from the Autonomous-Collaborator and Collaborator segments in their interactional behavior. For instance, Assertive-Collaborators use their health professionals as a source of health information significantly more often than Autonomous-Collaborators (*P*<.05) and find the health professional more useful than the internet compared with Collaborators (*P*<.05; see Multimedia Appendix 5 for more details).

Strong social connections are reflected in results showing Assertive-Collaborators rate significantly more highly than Collaborators on a range of interactions with others, including sharing personal experiences online and contacting someone online when they have problems with online health information (all *P* values<.05; see Multimedia Appendix 5 for more details). The frequency of their online information searches on behalf of friends and coworkers is also higher than Collaborators (all *P* values<.05). Finally, Assertive-Collaborators search online for health information for family members more frequently than Autonomous-Collaborators (*P*<.05).

Assertive-Collaborator patients are highly eHealth literate and are more likely to be chronically ill (*P*<.05; see Multimedia Appendix 4 for more details) and to have a relatively high number of visits to health care practitioners (compared with the Autonomous-Collaborator; *P*<.05; see Multimedia Appendix 3 for more details). Interestingly, the Assertive-Collaborator patient also feels that both communication effectiveness and the relationship with the health care professional have improved markedly, with the highest means on both of these outcome variables across all 4 segments (see Multimedia Appendix 3 for more details). Results show Assertive-Collaborators are the oldest of the baby boomer cohort (ie, First Wave boomers born between 1946 and 1949), widowed or permanently separated, and mostly US citizens (48/111, 43.2%; *P*<.05; see Multimedia Appendix 2 for more details).

#### Segment 4: Passives

This segment, the second largest group comprising 27.2% (271/996) of respondents, is made up of patients who typically are not collaborative, assertive, or autonomous in decision making involving health care practitioners. Of all 4 segments, patients who are passive decision makers rate online health information the least useful in comparison with information provided by a health practitioner. More explicitly, compared with the other 3 patient segments, Passives report the internet is significantly less useful as a source of information in their health-related decision making (*P*<.05; see Multimedia Appendix 5 for more details). Passive patients (compared with the other segments) are less likely to have looked online to try to diagnose a health condition themselves, to have researched a health-related product or service online, and/or to have signed up to receive email updates or alerts online (all *P* values<.05; see Multimedia Appendix 4 for more details).

Our analysis finds these Passive patients are less likely to have chronic health problems (*P*<.05), they have low eHealth literacy compared with the other segments (*P*<.05; see Multimedia Appendix 4 for more details), and they do not visit their health care practitioners often compared with the other segments (*P*<.05; see Multimedia Appendix 3 for more details). They also report, as a result of accessing health information online, the least effect on the quality of communication and the lowest levels of relationship quality with their health care professionals (*P*<.05; see Multimedia Appendix 3 for more details). In terms of demographic variables, segment 4 patients are most likely Third Wave boomers born between 1955 and 1959, are in a dissolved union, or never married. UK patients dominated this segment (116/271, 42.8%; *P*<.05; see Multimedia Appendix 2 for more details).

## Discussion

### Principal Findings

The overarching purpose of this study was to investigate more closely the decision-making styles of patients who seek online health information and to develop a typology of patients based on significant differences in those styles. Initially, we proposed 3 decision-making styles in relation to health professionals: collaborative, autonomous, and assertive. Data analysis confirmed these 3 patient decision-making styles. However, analysis revealed that patients’ decision making is considerably more complex than the 3 singular styles we proposed, with 2 combination styles of decision making and a fourth style distinguished by patient passivity. Specifically, to answer our first research question (see section Objectives), this research found patients can be clustered in 4 distinct segments; namely, Collaborators, combination Autonomous-Collaborators and Assertive-Collaborators, and Passive patients. In relation to the second research question, the research developed a typology of patients and described the segments based on similarities and differences in patient decision-making styles.

In summary, the major contributions of this paper include our identification of fundamental and distinct patient decision-making styles involving online health information, the typology of patients the empirical research revealed, and the multidimensional profiles (following) of those 4 patient segments developed on the basis of their decision making and other key practice-relevant variables.

#### Profile of Collaborators

Patients in the Collaborator segment are most likely to use the collaborative decision-making style and, according to the literature, participate with their practitioners in joint decision making. According to our data analysis, patients who are collaborative decision makers actively seek help from health care professionals, they use online health information to ask questions in interactions with their practitioners, and they have diagnoses they have made themselves confirmed by a professional (see Table 1). Applying previous findings (see the study by Essén et al [53]), Collaborators can be expected to be active partners in managing their health care, taking responsibility for finding and appraising relevant online health information, for disclosing their perspectives and preferences, and for weighing treatment options. As a result of sharing with their health care professionals the online health information they have accessed, these patients feel they can ask better questions and participate in discussion at a deeper level. Consistent with collaborative decision making, these patients review information and options together with their practitioners and thus are more involved in decision making [7,9,10,16,54]. In addition, according to our data, Collaborators are influenced by online health information to seek professional help, to have diagnoses they have made confirmed by their health professional, and to find out more from the practitioner about health conditions, treatment, and/or management.

Although a lower than average number of these patients live with chronic health problems, collaborative decision makers visit their health professionals more often. Some researchers explain this distinctive pattern as being motivated by the patients wanting to treat conditions promptly and avoid further complications [55]. However, others warn that such behavior can be problematic when it results in overuse of the health care system [56]. Patients in the Collaborator segment are moderately eHealth literate compared with Autonomous-Collaborators and Assertive-Collaborators (both segments highly eHealth literate) and passives (low eHealth literacy). Building on the work by Schulz et al [28], more research could further investigate the relationships between chronic health problems, visits to health care professionals, and eHealth literacy. For example, 1 important clinical implication is that raising the eHealth literacy of patients in the Collaborators segment could enable more self-responsibility thereby reducing overdependence on health professionals and unnecessary visits to those practitioners.

#### Profile of Autonomous-Collaborators

The Autonomous-Collaborator segment includes patients who have accessed online health information and use a combination of decision-making styles: they are both autonomous and collaborative in their decision making with health professionals. Thus, at times, they make decisions in collaboration with practitioners (see section Segment 1 profile above), and at other times, they make decisions independently of health care practitioners; for instance, they try to diagnose a health condition or disease they or someone they know has, and they will also treat a health condition or disease without help from a health professional (Table 2).

When patients in this segment use autonomous decision making, they are self-active in addressing their health needs [7,16]. For example, autonomous decision makers access health information online to understand a specific condition, to explore whether symptoms are related to clinically meaningful diseases, and to diagnose and treat health conditions themselves [5,16]. In other words, patients practicing the Autonomous-Collaborative decision-making style will make autonomous decisions regarding their health at times without consulting practitioners.

Internet-informed patients can possess both knowledge and treatment preferences before they interact with practitioners; therefore, they are better equipped to take a fuller and more participatory role in decision making. Thus, in their interactions with health care professionals, these patients can choose to share information to be interpreted, evaluated, contextualized, and deliberated as part of collaborative decision making with practitioners. However, at other times, Autonomous-Collaborative decision makers will decide not to share their online health information; yet it is likely that having informed themselves before the interaction they feel more empowered in their decision making because they are better able to understand what the practitioner says or to trust the practitioner when the online information verifies the practitioner’s explanation.

This single segment contains all respondents who practice autonomous decision making; however, the results clearly show that at times, these patients prefer to make joint decisions with practitioners. Similar to patients in the Assertive-Collaborator segment, patients who combine autonomous and collaborative decision making are highly eHealth literate. However, there are important differences between these 2 segments in terms of how the online health information they access influences patient decision making with health professionals and the frequency of visits to practitioners. Assertive-Collaborators typically challenge their practitioners based on the online information, whereas Autonomous-Collaborators incorporate the online information they have accessed independently in interactions with practitioners. In other words, although results indicate this segment uses online information to make decisions independently of health professionals (autonomous decision-making style), data show they collaborate and engage when they do consult practitioners. Interestingly, Assertive-Collaborators are more likely to have chronic health problems, which might explain the high number of visits to their health professionals. A question that arises here is whether the assertive decision-making style is impacted by chronic health problems and the potential frustration associated with being persistently sick. Future research could further investigate those relationships.

At a time when shared decision making and self-responsible/self-managing patients are seen as key to reducing the costs of health care service provision, Autonomous-Collaborators are clearly a desirable segment. Certainly, their collaborative behaviors are likely to make this segment efficient for health professionals to serve. These patients are likely to be easy and fast to work with; therefore, relational labor costs for the practitioner will be minimal. Therefore, from the perspective of health care policy makers, this segment (predominantly UK Fourth Wave baby boomers born between 1960 and 1964) will impose the smallest financial cost on the health care system. Future research on the implications of these findings for clinicians could identify and explain the reasons some patients are assertive and others are autonomous in relation to their practitioners when making decisions.

#### Profile of Assertive-Collaborators

The Assertive-Collaborator segment comprises patients who will be both assertive and collaborative in decision making with practitioners. Thus, some of their health care decisions are collaborative in that these patients participate actively with their health care professionals in joint decisions on treatment and health management. However, these same patients are also self-determined decision makers who change, refuse, or discontinue recommended treatment and switch health professionals (Table 2).

All patients who are likely to be assertive and/or challenging with online health information belong to this specific segment. When the online health information they have sourced differs from that provided by practitioners, Assertive-Collaborators can use that information to challenge or oppose the opinions or recommendations of their practitioners and assert their own preferences for health care treatment. In line with previous research, patients in this segment can be expected to have contrary views to their practitioners, contradict practitioner interpretations of their health situation, and/or insist on tests and treatments based on online health information they have gathered [16]. When patients are misinformed by online health information, these tests and treatments are likely to be inappropriate. Conversely, when patients are better informed than practitioners, their health-related requests may well be appropriate. Finally, when a patient chooses to raise accurate online health information that conflicts with their practitioner’s opinions, that decision can threaten the professional authority of the health care professional [22]. In summary, assertive patients can be challenging—sometimes confrontational—in their decision making with practitioners. In line with the literature [7], this research used the following as indicators of assertive patient responses: changing the treatment recommended by a health care practitioner, refusing or discontinuing recommended treatment, and changing from one professional to another.

At other times, these same patients are also collaborative in their decision making with practitioners. Therefore, an important clinical implication of these results is while their health professionals can be confident that this segment will challenge them at times, practitioners can also expect, in other circumstances, this segment will engage in joint decision making in which health information sourced online is shared and deliberated during the interaction. Health professionals can also be encouraged by the fact that these patients are displaying assertive decision-making behaviors inside the relationship rather than independently of the practitioner. This indicates that the nature of the relationship allows for debate, and that while online health information is important to the patient in their decision-making, so too is the practitioner.

Clinical implications for the Assertive-Collaborator segment include ensuring these patients have access to high-quality health information and that they maintain high eHealth literacy (the mean eHealth literacy of this segment is 2.19, highest shared with Autonomous-Collaborators). These strategies could reduce the potential for this segment to be misinformed, leading to confrontational interactions and decision making in the context of the patient-practitioner relationship, thus avoiding the possibilities of adverse health effects for the patient and of patient switching for the practitioner. Health professionals also could be trained in managing interactions with these patients so the online health information they share with practitioners is discussed and considered in a respectful open dynamic. In this way, practitioners’ acknowledgment of their patients’ rights and preferences in seeking and sharing online information would support such information activity as an integral part of patients taking more responsibility for their own health. For example, as a basis for shared decision making, early in each encounter, the practitioner could encourage the patient to talk about any health information they have accessed online preconsultation.

In addition, health professionals could be trained in conflict management and resolution so that the characteristic debate of Assertive-Collaborators’ decision making can be vigorous without compromising the safety of the patient-practitioner relationship in which it occurs. This is the only segment that contains potentially confrontational patients; future studies should investigate the health-related situations in which this segment is most likely to be assertive and those when patients will be collaborative with health professionals. Finally, most of this segment is likely to suffer from a chronic health condition; more research is warranted on the links between specific health problems, online health information, and the decision making of Assertive-Collaborators.

#### Profile of Passives

Patients in the Passive segment characteristically are not influenced significantly by online health information to be collaborative, autonomous, or assertive in their decision making with health professionals. Instead, this group is characteristically passive, a finding consistent with previous work finding some patients prefer to rely on the traditional paternalistic patient-physician relationship and leave decision making to their practitioners [14,18]. Patients in this segment visit their health professionals the least frequently of all 4 segments in the typology. This segment (predominantly First Wave baby boomers from the United Kingdom) could be regarded as a risk group because coupled with the negligible impact of internet-accessed health information, the low number of health professional visits means there is the risk that serious conditions are undetected. Given the low influence of online health information on the decision making of this sizeable segment, policy makers and practitioners urgently need to identify those communication channels that are most effective in reaching and supporting these patients. Future research should establish the interaction and communication preferences of this segment to further develop the clinical implications for this segment.

### Contributions

To conclude this section, it is worth noting that patients in both the Collaborator segment and the Assertive-Collaborator segment report chronic health problems. In addition, patients in both segments make more visits to their health care practitioners. Yet, the Assertive-Collaborator is significantly more eHealth literate than the Collaborator, suggesting a link between eHealth literacy and more assertive patient behaviors in interactions with health care practitioners (as behavioral dimensions of patient empowerment). These results are in line with the recent findings of Schulz et al [28] that the number of visits to a health care professional is independent of eHealth literacy, and there is a significant positive relationship between high eHealth literacy and empowerment. For relatively healthy people, high eHealth literacy is linked with being both autonomous and collaborative in decision making with practitioners and with a medium number of practitioner visits (see section Profile of Autonomous-Collaborators). In contrast, healthy patients with lower levels of eHealth literacy are unaffected by online health information and have low frequency of visits to health care practitioners (see section Profile of Passives).

In addition, it is important to note that the majority of patients who use online health information in their decision making with health care professionals are characteristically collaborative to some degree, as reflected in the segment titles (Collaborators, Autonomous-Collaborators, and Assertive-Collaborators). For the 72.8% (725/996) of respondents in these 3 segments, collaboration with their practitioners is an important interactional behavior. In other words, the majority of patients show significant collaboration in their decision making with health care professionals. However, at times, 1 distinguishable group of that majority prefers to exercise individual autonomy in their decision making, and another group prefers to be assertive with their health professionals. In addition, this study finds that a substantial number of patients adopt a distinctly passive decision-making style (271/996, 27.2%) with practitioners.

Thus, the research makes 3 main contributions to the literature. First, it increases our understanding of online health information and patient decision making by identifying in the literature and exploratory research 3 patient decision-making styles: collaborative, autonomous, and assertive. Second, the research extends the literature on patient segments [57] by deriving a typology based on decision-making styles, additional sociodemographic and behavioral characteristics, and outcome variables. The 4 segments are profiled as the Collaborators, the Autonomous-Collaborators, the Assertive-Collaborators, and the Passive patients. Third, the research demonstrates that collaborative decision-making is dominant among patients, either in its pure form (229/996, 23.0%) or in combination with autonomous (385/996, 38.7%) or assertive decision making (111/996, 11.1%).

### Limitations

This study, as any other study, has some limitations that offer opportunities for future research. First, the purpose of conducting this research in multiple countries was to identify shared patterns in patient decision making across the United Kingdom, the United States, and New Zealand. However, to allow for differences in data from the 3 countries, we included *country of residence* as one of the passive variables in our analysis. As discussed, we found significant associations between the country of residence of our respondents and the segments they belong to. We believe this considerably strengthens the contribution of our research. We also acknowledge that in this research, we cannot make any conclusions beyond the 3 western countries we investigated. This is important to note as patients in nonwestern countries with different core cultural values might have differences in their use of internet-sourced health information and patient decision making. More research is needed to replicate or identify differences across different cultures. Next, this research uncovered general patient decision-making styles. However, there might be numerous contextual factors that influence actual decision making in a specific context. Future research could investigate those factors and their impact on behavior (or a good proxy for that). In our research we deliberately focused on online information and did not account for other sources of health information (ie, books). It would be interesting to further investigate whether the same decision-making styles and typology emerge if patients use more traditional health information sources. In addition, this study looked at the behavior and decision making from the patient’s perspective. One very interesting avenue for future research is to investigate how practitioners perceive different patient segments and how practitioner behaviors impact such segments. In addition, our sample was baby boomers, certainly an important cohort because of the effects on society of managing their health care; however, the typology presented here should be investigated using patients from other age groups. Furthermore, the research sample was fairly homogenous. Therefore, the results and their generalizability should be considered in light of the homogenous sample we have. Moreover, because we used cross-sectional data to study patients’ decision-making styles, our research design did not account for the dynamic of variables. Moreover, we relied on self-reported outcomes that were not objectively measured; that is, they were not a measurement of how patients actually engage physicians during consultations. Finally, the way the survey was conducted might have influenced the results. A longitudinal study with real live data would be very useful as a follow-up. We believe that this research opens up a wide range of possibilities for future research into decision making and patient-practitioner interactions.

### Conclusions

This research demonstrates complex differences regarding the decision making, online health information, and interactional behaviors of baby boomer patients. Close investigation of the variations in characteristics showed that according to their decision-making style, patients experience significant differences in the way online health information impacts their interactions with health professionals. The typology of patients based on their decision making provides a more sophisticated framework than simplistic descriptors (eg, *empowered* or *nonempowered* patients) or demographic characteristics (eg, age or nationality) for distinguishing practice-relevant and addressable segments of patients. Understanding these segments and their clinical implications will enable practitioners and policy makers to implement health care communication programs that are meaningful and valued by different groups of patients, thereby supporting more accurate targeting and more successful outcomes for health care services in the long term.

## Appendix

Multimedia Appendix 1 Questions/items for online domain and online information outcome variables.

Multimedia Appendix 2 Segments described by demographic variables.

Multimedia Appendix 3 Segments described by online information outcome variables.

Multimedia Appendix 4 Segments described by health-related domain variables.

Multimedia Appendix 5 Segments described by online domain variables.

## Abbreviations

AVE: average variance extracted
CFA: confirmatory factor analysis
CR: compositional reliability
eHealth: electronic health
