# Supplementary material for: A Typology of Patients Based on Decision-Making Styles: Cross-Sectional Survey Study
Source: J Med Internet Res. 2019 Nov 20;21(11):e15332. doi: 10.2196/15332 (PMC6893560; doi:10.2196/15332)
Supplement: Multimedia Appendix 1 [file jmir_v21i11e15332_app1.docx]

Appendix A. Questions/items for online domain and online information outcome variables

| Items | Scale (coded as) |
| --- | --- |
| **Online Domain Variables** |  |
| **Health information sources - health professional vs internet** |  |
| In general, when looking for health-related information, I used the following sources: |  |
| Health professional | 1 (Never) – 5 (Very often) |
| Internet | 1 (Never) – 5 (Very often) |
|  |  |
| In comparison to information provided by a *health professional*, how useful has the health information been that you found *online* over the last 6 months? | 1 (Much less useful)  – 5 (Much more useful) |
|  |  |
| **Frequency of online health-related behaviours** |  |
| Please click the button that completes each statement: In the last 6 months... |  |
| I looked online to try to diagnose a health condition | 1 (Never) – 5 (Very often) |
| I researched a health-related product or service online | 1 (Never) – 5 (Very often) |
| I signed up to receive email updates or alerts about health-related issues | 1 (Never) – 5 (Very often) |
| I read or watched someone else’s commentary or experience online about health-related issues | 1 (Never) – 5 (Very often) |
| I went online to find others who might have health concerns similar to mine | 1 (Never) – 5 (Very often) |
| I shared my own personal health experience online in some way | 1 (Never) – 5 (Very often) |
| I posted a comment or review online about a health-related product, service, or person | 1 (Never) – 5 (Very often) |
| I rated a health-related product, service, or person | 1 (Never) – 5 (Very often) |
|  |  |
| **Responses to problems with online health information** |  |
| Please click the button that completes each statement:   In the last 6 months, if I had problems with the health information I found online |  |
| I talked with a friend about the information | 1 (Never) – 5 (Very often) |
| I talked with a health professional about the information | 1 (Never) – 5 (Very often) |
| I contacted someone else online | 1 (Never) – 5 (Very often) |
|  |  |
| **Online Information Outcome Variables** |  |
| In general, as a result of searching for health information online… |  |
| I can **communicate** more effectively with my health professional(s) | 1 (Strongly disagree)  – 5 (Strongly agree) |
| The quality of the **relationship** with my health professional(s) has improved | 1 (Strongly disagree)  – 5 (Strongly agree) |
