# Supplementary material for: A Typology of Patients Based on Decision-Making Styles: Cross-Sectional Survey Study
Source: J Med Internet Res. 2019 Nov 20;21(11):e15332. doi: 10.2196/15332 (PMC6893560; doi:10.2196/15332)
Supplement: Multimedia Appendix 2 [file jmir_v21i11e15332_app2.docx]

Appendix E: Segments described by demographic variables

|  | Total  Sample | | | Segment 1  Collaborators | | Segment 2  Autonomous-Collaborators | | Segment 3  Assertive- Collaborators | | Segment 4  Passives | |  |
| --- | --- | --- | --- | --- | --- | --- | --- | --- | --- | --- | --- | --- |
| Demographic variables | n | | % | n | % | n | % | n | % | n | % | X2 |
| **Country** |  | |  |  |  |  |  |  |  |  |  |  |
| UK | 407 | | 40.9 | 73 | 31.9 | 189 | 49.1 | 29 | 26.1 | 116 | 42.8 | 36.19^a^ |
| US | 313 | | 31.4 | 80 | 34.9 | 115 | 29.9 | 48 | 43.2 | 70 | 25.8 |  |
| NZ | 276 | | 27.7 | 76 | 33.2 | 81 | 21 | 34 | 30.6 | 85 | 31.4 |  |
| **Relationship status** | |  |  |  |  |  |  |  |  |  |  |  |
| Never been married/legally registered | 177 | | 17.8 | 31 | 13.5 | 78 | 20.3 | 18 | 16.2 | 50 | 18.5 | 32.039 ^a^ |
| Divorced/dissolved union | 176 | | 17.7 | 35 | 15.3 | 70 | 18.2 | 20 | 18 | 51 | 18.8 |  |
| Widow/widower | 56 | | 5.6 | 19 | 8.3 | 13 | 3.4 | 8 | 7.2 | 16 | 5.9 |  |
| Permanently separated | 36 | | 3.6 | 12 | 5.2 | 12 | 3.1 | 5 | 4.5 | 7 | 2.6 |  |
| Legally married | 533 | | 53.5 | 129 | 56.3 | 197 | 51.2 | 60 | 54.1 | 147 | 54.2 |  |
| Legally registered in civil union | 18 | | 1.8 | 3 | 1.3 | 15 | 3.9 | 0 | 0 | 0 | 0 |  |
|  |  | |  |  |  |  |  |  |  |  |  |  |
| **Age** |  | |  |  |  |  |  |  |  |  |  |  |
| 1946-49 First Wave | 167 | | 16.8 | 47 | 20.5 | 41 | 10.6 | 22 | 19.8 | 57 | 21 | 33.858 ^a^ |
| 1950-54 Second W. | 260 | | 26.1 | 64 | 27.9 | 102 | 26.5 | 32 | 28.8 | 62 | 22.9 |  |
| 1955-59 Third W. | 256 | | 25.7 | 54 | 23.6 | 90 | 23.4 | 27 | 24.3 | 85 | 31.4 |  |
| 1960-64 Fourth W. | 313 | | 31.4 | 64 | 27.9 | 152 | 39.5 | 30 | 27 | 67 | 24.7 |  |
| Note: ^a^ *P* < = 0.05, ^b^ *P* < .1. Income, Education, Gender are not significant *P* > .1. | | | | | | | | | | | | |
