# Supplementary material for: A Typology of Patients Based on Decision-Making Styles: Cross-Sectional Survey Study
Source: J Med Internet Res. 2019 Nov 20;21(11):e15332. doi: 10.2196/15332 (PMC6893560; doi:10.2196/15332)
Supplement: Multimedia Appendix 3 [file jmir_v21i11e15332_app3.docx]

Appendix B. Segments described by online information outcome variables

|  | Total Sample | | Segment 1 | | Segment 2 | | Segment 3 | | Segment 4 | |  |  |
| --- | --- | --- | --- | --- | --- | --- | --- | --- | --- | --- | --- | --- |
|  |  |  | Collaborators | | Autonomous-Collaborators | | Assertive-Collaborators | | Passives | |  |  |
|  | M | SD | M | SD | M | SD | M | SD | M | SD | F value | Post hoc test |
| HP visits | 3.29 | 1.70 | 3.89 | 1.77 | 3.24 | 1.60 | 3.9 | 1.58 | 2.6 | 1.55 | 32.76 ^a^ | 4-1,2,3^a^  2-1/3 ^a^ |
| Effectiveness of communication | 3.62 | 0.87 | 3.93 | 0.66 | 3.66 | 0.83 | 4.0 | 0.7 | 3.1 | 0.93 | 57.24 ^a^ | 4-1,2,3 ^a^;  3-2 ^a^;  2-1 ^a^ |
| Quality of relationship | 3.29 | 0.89 | 3.50 | 0.84 | 3.4 | 0.79 | 3.65 | 0.89 | 2.8 | 0.87 | 41.78 ^a^ | 4-1,2,3 ^a^;  3-2^b^ |

Notes: Significant at: ^a^ *P* < .05; ^b^ *P* < .1, Health professional (HP) visits: higher values represent higher frequency (1=“None”, 7=“10 or more”).
