# Supplementary material for: A Typology of Patients Based on Decision-Making Styles: Cross-Sectional Survey Study
Source: J Med Internet Res. 2019 Nov 20;21(11):e15332. doi: 10.2196/15332 (PMC6893560; doi:10.2196/15332)
Supplement: Multimedia Appendix 4 [file jmir_v21i11e15332_app4.docx]

Appendix C. Segments described by health-related domain variables

|  | Total Sample | | Segment 1 | | Segment 2 | | Segment 3 | | Segment 4 | |  |  |
| --- | --- | --- | --- | --- | --- | --- | --- | --- | --- | --- | --- | --- |
|  |  |  | Collaborators | | Autonomous-Collaborators | | Assertive-Collaborators | | Passives | |  |  |
|  | n | % | n | % | n | % | n | % | n | % | X2 |  |
| Chronic health problems^c^ | | | | | | |  |  |  |  |  |  |
| No | 261 | 26.2 | 47 | 20.5 | 105 | 27.3 | 17 | 15.3 | 92 | 33.9 | 19.258^a^ |  |
| Yes | 735 | 73.8 | 182 | 79.5 | 280 | 72.7 | 94 | 84.7 | 179 | 66.1 |  |  |
|  | M | SD | M | SD | M | SD | M | SD | M | SD | F value | Post hoc test |
| eHealth literacy^d^ | | | | | |  |  |  |  |  |  |  |
|  | 3.68 | 0.64 | 3.67 | 0.63 | 3.8 | 0.59 | 3.81 | 0.58 | 3.47 | 0.67 | 17.191^b^ | 4-1/2/3 ^a^; 1-2^b^ |

Notes: Significant at: ^a^ *P* < .05; ^b^ *P* < .1, ^c^Respondents were labelled as having Chronic health problem if indicated that they have at least one of the following chronic health problems: Diabetes. High cholesterol. High blood pressure. Asthma, bronchitis, emphysema, or other lung conditions. Osteoarthritis. Osteoporosis. Heart disease, heart failure, or heart attack. Stroke. Mental health/Neurological conditions e.g., depression, substance abuse, dementia, etc. Cancer or Other chronic condition .^d^eHealth literacy is coded on a five-point scale; “Strongly disagree” (1) to “Strongly agree” (5)- assessed using the eHealth Literacy Scale.
