# Supplementary material for: A Typology of Patients Based on Decision-Making Styles: Cross-Sectional Survey Study
Source: J Med Internet Res. 2019 Nov 20;21(11):e15332. doi: 10.2196/15332 (PMC6893560; doi:10.2196/15332)
Supplement: Multimedia Appendix 5 [file jmir_v21i11e15332_app5.docx]

Appendix D. Segments described by online domain variables

|  | Total Sample | | Segment 1 | | | Segment 2 | | Segment 3 | | | Segment 4 | |  |  |
| --- | --- | --- | --- | --- | --- | --- | --- | --- | --- | --- | --- | --- | --- | --- |
|  |  |  | Collaborators | | | Autonomous-Collaborators | | Assertive-Collaborators | | | Passives | |  |  |
|  | M | SD | | M | SD | M | SD | M | SD | | M | SD | F value | Post hoc test |
|  | | | | | | | | | |  |  |  |  |  |
| **Health-related information source - health professional (HP) vs internet** | | | | | | | | | | | | | | |
| Health professional | 3.71 | 1.05 | | **4.13** | 0.82^a^ | **3.58** | 1.05 ^a^ | 4.00 | | 0.86 | 3.42 | 1.15 | 24.925 | 4-1,3^a^;1-2 ^a^; 2-3 ^a^ |
| Internet | 3.69 | 1.01 | | **3.64** | 0.91 | **3.83** | 1.05 | 3.89 | | 0.89 | 3.44 | 1.05 | 9.59 | 4-2,3 ^a^ |
|  |  |  | |  |  |  |  |  | |  |  |  |  |  |
| Usefulness HP vs internet | 3.22 | 0.92 | | 3.18 | 0.84 | 3.32 | 0.95 | 3.55 | | 0.78 | 2.98 | 0.95 | 13.15 ^a^ | 4-2,3 ^a^; 1-3 ^a^ |
|  |  |  | |  |  |  |  |  | |  |  |  |  |  |
| **Frequency of search for other people** | | | | | | |  |  | |  |  |  |  |  |
| Family member | 2.18 | 1.13 | | 2.18 | 1.03 | 2.38 | 1.15 | 2.87 | | 1.24 | 1.75 | 0.98 | 21.79 | 4-1,,2, 3 ^a^; 1-3 ^a^ |
| Friend | 1.6 | 0.93 | | 1.6 | 0.91 | 1.86 | 1.02 | 2.02 | | 0.92 | 1.35 | 0.69 | 20.62 | 4-1,,2, 3 ^a^; 1-2, 3 ^a^ |
| Co-worker | 1.36 | 0.76 | | 1.25 | 0.59 | 1.51 | 0.9 | 1.62 | | 0.93 | 1.14 | 0.44 | 15.11 | 4-,2, 3 ^a^; 1-2, 3 ^a^ |
|  |  |  | |  |  |  |  |  | |  |  |  |  |  |
|  |  |  | |  |  |  |  |  | |  |  |  |  |  |
| **Frequency of online health-related behaviours** | | | | | | | | | | | | | | |
| To diagnose health condition | 2.85 | 1.09 | | 2.9 | 0.97 | 3.14 | 1.1 | 3.14 | | 1.09 | 2.3 | 0.97 | 38.76 | 4-1,2,3 ^a^;  1-2 ^a^ |
| To sign up for email updates or alerts | 1.89 | 1.17 | | 1.82 | 1.12 | 2.02 | 1.23 | 2.51 | | 1.32 | 1.49 | 0.9 | 24.21 | 4-1,2,3 ^a^;1-3 ^a^; 2-3 ^a^ |
| To read or watch someone else’s commentary | 2.16 | 1.16 | | 2.15 | 1.06 | 2.43 | 1.21 | 2.51 | | 1.14 | 1.65 | 0.95 | 31.36 | 4-1,2,3 ^a^; 1-2,3 ^a^ |
| To find others with similar | 2.05 | 1.20 | | 1.93 | 1.10 | 2.36 | 1.28 | 2.49 | | 1.29 | 1.52 | 0.89 | 35.37 | 4-1,2,3 ^a^; 1-2,3 ^a^ |
| To share own health experience | 1.58 | 1.00 | | 1.47 | 0.89 | 1.77 | 1.12 | 1.99 | | 1.25 | 1.21 | 0.57 | 26.29 | 4-1,2,3 ^a^; 1-2,3 ^a^ |
| To post a comment or review | 1.51 | .97 | | 1.43 | 0.87 | 1.68 | 1.08 | 1.88 | | 1.27 | 1.18 | 0.54 | 21.39 | 4-1,2,3 ^a^; 1-2,3 ^a^ |
| To rate product, service, person | 1.55 | 0.99 | | 1.41 | 0.84 | 1.77 | 1.11 | 1.98 | | 1.25 | 1.2 | 0.58 | 28.69 | 4-2,3 ^a^; 1-2,3 ^a^ |
|  |  |  | |  |  |  |  |  | |  |  |  |  |  |
| **Responses to problems with online health information** | | | | | | | | | | | | | | |
| I talked with a friend | 2.01 | 1.1 | | 1.92 | 0.98 | 2.24 | 1.15 | 2.51 | | 1.13 | 1.55 | 0.9 | 33.34 | 4-2,3 ^a^; 1-2,3 ^a^ |
| I talked with a health professional | 2.44 | 1.24 | | 2.88 | 1.22 | 2.52 | 1.2 | 3.15 | | 1.06 | 1.65 | 0.96 | 72.12 | 4-2,3 ^a^; 1-2 ^a^, 2-3 ^a^ |
| I contacted someone else online | 1.47 | 0.56 | | 1.32 | 0.68 | 1.65 | 0.97 | 1.84 | | 1.09 | 1.17 | 0.55 | 27.22 | 4-2,3 ^a^; 1-2,3 ^a^ |

Notes: Significant at: ^a^ *P* < .05. See Appendix A for full question and scale.
